# Safety and efficacy of regional citrate anticoagulation for continuous renal replacement therapy in liver failure patients: a systematic review and meta-analysis

**DOI:** 10.1186/s13054-019-2317-9

**Published:** 2019-01-24

**Authors:** Wei Zhang, Ming Bai, Yan Yu, Lu Li, Lijuan Zhao, Shiren Sun, Xiangmei Chen

**Affiliations:** 1 The Nephrology Department of Xijing Hospital, the Fourth Military Medical University, No. 127 Changle West, Road, Xi’an, 710032 Shaanxi China; 2 State Key Laboratory of Kidney Disease, Department of Nephrology, Chinese People’s Liberation Army General Hospital and Military Medical Postgraduate College, 28th Fuxing Road, Beijing, 100853 China

**Keywords:** Anticoagulation, Citrate, Liver failure, Continuous renal replacement therapy

## Abstract

**Background:**

Regional citrate anticoagulation (RCA) is a widely used strategy for continuous renal replacement therapy (CRRT). Most of the current guidelines recommend liver failure as one of the contraindications for citrate anticoagulation. However, some studies suggested that the use of citrate for CRRT in liver failure patients did not increase the risk of citrate-related complications. The purpose of this systematic review is to summarize the current evidences on the safety and efficacy of RCA for CRRT in liver failure patients.

**Methods:**

We performed a comprehensive search on PubMed, Embase, and the Cochrane Library databases from the inception to March 1, 2018. Studies enrolled adult (age > 18 years) patients with various levels of liver dysfunction underwent RCA-CRRT were included in this systematic review.

**Results:**

After the study screening, 10 observational studies with 1241 liver dysfunction patients were included in this systematic review. The pooled rate of citrate accumulation and bleeding was 12% [3%, 22%] and 5% [2%, 8%], respectively. Compared with the baseline data, the serum pH, bicarbonate, and base excess (BE), the rate of metabolic alkalosis, the serum ionized calcium (ionCa) and total calcium (totCa) level, and the ratio of total calcium/ionized calcium (totCa/ionCa) significantly increased at the end of observation. However, no significant increase was observed in serum citrate (MD − 65.82 [− 194.19, 62.55]), lactate (MD 0.49 [− 0.27, 1.26]) and total bilirubin concentration (MD 0.79 [− 0.70, 2.29]) at the end of CRRT. Compared with non-liver failure patients, the live failure patients showed no significant difference in the pH (MD − 0.04 [− 0.13, 0.05]), serum lactate level (MD 0.69 [− 0.26, 1.64]), and totCa/ionCa ratio (MD 0.03 [− 0.12, 0.18]) during CRRT. The median of mean filter lifespan was 55.9 h, with a range from 22.7 to 72 h.

**Conclusions:**

Regional citrate anticoagulation seems to be a safe anticoagulation method in liver failure patients underwent CRRT and could yield a favorable filter lifespan. Closely monitoring the acid base status and electrolyte balance may be more necessary during RCA-CRRT in patients with liver failure.

**Electronic supplementary material:**

The online version of this article (10.1186/s13054-019-2317-9) contains supplementary material, which is available to authorized users.

## Background

Regional citrate anticoagulation (RCA) has become a widely used strategy for continuous renal replacement therapy (CRRT) [1]. The advantages of citrate anticoagulation include the reduction of bleeding risk and extension of extracorporeal circuit lifespan [2]. However, most of current evidences on the use of citrate anticoagulation were limited in patients without liver failure. For liver failure patients, the risk of citrate accumulation might be potentially increased because of the impaired citrate metabolism in the citric acid cycle, which is mainly processed in the liver [3–5].

Currently, the Kidney Disease Improving Global Outcomes (KDIGO) organization recommends citrate as the first-line anticoagulation agent over heparin for CRRT in patients without citrate contraindication. In this clinical practice guideline, severe liver failure was listed as one of the contraindications of citrate anticoagulation, regarding the potential citrate accumulation and the subsequent metabolic complications [6]. In the Chinese Standard Operating Procedure for blood purification, severe liver failure was listed as one of the contraindications of citrate anticoagulation for CRRT as well [7]. Link et al. reported that citrate accumulation correlated to the hepatic clearance and was an independent risk factor for 28-day mortality [8]. However, several prospectively designed studies suggested that RCA could be safely and effectively used for CRRT in critically ill patients with liver dysfunction [9–11].

There are controversial results on the feasibility of RCA in patients with liver failure among the published literatures. Therefore, we performed this systematic review to evaluate the safety and efficacy of RCA-CRRT in patients with liver failure.

## Method

### Search strategy

We performed a comprehensive search on PubMed, Embase, and Cochrane Library databases by using the MeSH terms: liver failure, renal replacement therapy, citrate, and anticoagulants. The searching was performed on 1 March 2018. The MeSH terms and entry terms from PubMed were also used in the search of Embase and Cochrane Library. Furthermore, we manually searched the reference list of the retrieved studies and review articles for additional publications. There was no language restriction in the searching.

### Study selection

Studies with the following criteria were considered for inclusion: (i) studies that included adult (age > 18 years) patients with various levels of liver dysfunction and (ii) all patients underwent CRRT and the anticoagulation strategy was RCA. Studies with the following characteristics were excluded: (i) full text was not available, (ii) studies that did not analyze the safety and efficacy of RCA in patients with liver failure, (iii) no data on the safety and efficacy of RCA was available, (iv) sample size ≤ 5, and (v) the following article styles: review articles, case reports, letters, editorials, conference abstracts, and comments.

### Study quality

A modified version of the Newcastle-Ottawa Scale [12] was used to assess the quality of the included studies (Additional file 1: Table S1). The scale includes eight items that evaluate three aspects of quality: subject selection, comparability of cohorts, and assessment of outcomes. Study with a total score of 6–8, 4–5, and ≤ 3 was considered as high, moderate, and low quality, respectively.

### Data collection

All criteria and needed data were defined before the study screening and data collection. For study screening, the article type, title, and abstract were assessed first. Thereafter, the full texts of the papers passed initial screening were reviewed for the final exclusion. Furthermore, the following information of the included papers was recorded: (i) origin of the article, (ii) geographic origin, (iii) study type, (iv) inclusion and exclusion criteria, (v) information relevant to the quality of the study, (vi) baseline data of patients, (vii) liver failure relevant data, and (viii) information relevant to the safety and efficacy of RCA. Parameters only illustrated in graph were extracted by using the Engauge Digitizer software (version 9.8,© 2014 Mark Mitchell).

### Statistical analysis

For continuous variables, median (IQR interquartile range) was converted to mean ± SD (standard deviation), according to the methods reported by Wan et al. [13]. For categorical variables, the incidence was calculated by dividing the number of total patients by the number of observed events. Mean and SD across studies were combined according to the formula reported in the Cochrane Handbook [14].

MD (mean difference) and RD (risk difference) were pooled to evaluate the difference between the end of observation and the start of CRRT for continuous variables and categorical variables, respectively. Heterogeneity of the included studies was assessed by *I*^2^ statistic. The source of heterogeneity was explored by sequentially excluding the included studies. Subgroup analysis was conducted when the patients were grouped according to the severity of liver failure. The MD and RD were pooled using Review Manager (RevMan) [Computer program]. Version 5.3. Copenhagen: The Nordic Cochrane Centre, the Cochrane Collaboration, 2014. The incidences of the outcomes were pooled using R software (version 3.5.1,© 2018 the R Foundation for Statistical Computing). A *P* value less than 0.05 was considered as a statistical significance. *P* value was assessed by using random effects model for *I*^2^ > 50% and fixed effects model for *I*^2^ ≤ 50% [15].

## Results

### Selection of studies

**Fig. 1:**
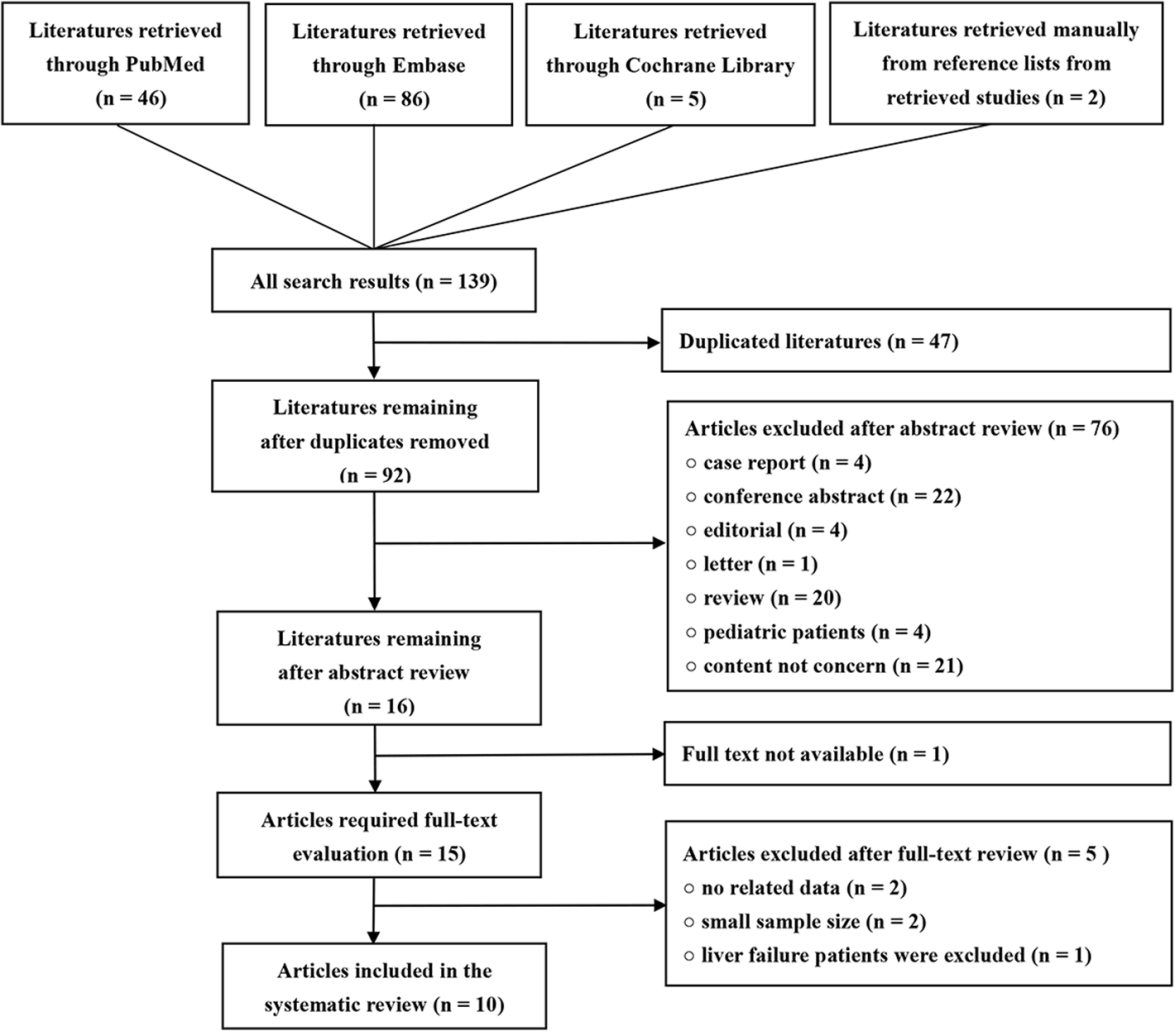
Study inclusion flow chart

The study inclusion flow chart is showed in Fig. 1. After the searching, 139 references were identified. Of the identified references, 47 and 51 were excluded because of duplications and article types, respectively. Furthermore, 21 papers were excluded because they did not assess the safety and efficacy of RCA-CRRT in liver failure patients, and 4 papers were excluded because their included patients were < 18 years old. At last, 15 articles underwent full-text reviewing, 2 [16, 17] of which were excluded because their sample sizes were less than 5. Additionally, 3 studies were excluded because the needed data were not reported. The full text of one study was not available after searching several databases and local libraries. Additionally, we got no responses after sending several letters to the authors of this paper. Finally, 10 studies [9–11, 18–24] with 1411 patients were included in this systematic review.

### Description of the included studies and patients

The characteristics of the 10 included studies are showed in Table 1. All of the included studies were observationally designed. And, 70% of the included studies enrolled retrospective cohort. The detailed protocols of RCA-CRRT in these studies are showed in Table 2. The model of CRRT was CVVHD in 5 (50%) studies, CVVHDF in 2 (20%) studies, and SLED in 1 (10%) study. And the model of CRRT was not reported in the remaining 2 (20%) studies. Five (50%) studies [9, 10, 18, 20, 21] conducted comparisons between different time points during RCA-CRRT (including 0–72 h, 0–24 h, 1–5 days, 1–7 days, and 0–7 days). Four (40%) studies [11, 19, 22, 24] compared data across groups divided by liver function. And two studies [23, 24] compared data between the groups with or without citrate accumulation.

**Table 1:** Characteristics of the included studies

Source	Design	Setting	Exclusion	Patients	End-points
Schultheiss et al. [9] (GER, 2012)	POS	Medical ICU	pH > 7.55 or < 7.1; ionCa < 0.9 mmol/l	Cirrhosis; acute LF	Circuit lifetime; acid-base status; electrolyte balance; citrate accumulation
Lahmer et al. [10] (GER, 2015)	POS	Medical ICU	pH > 7.55 or < 7.1; ionCa < 0.9 mmol/l	Cirrhosis; acute LF	Acid-base status; electrolyte balance; serum citrate level
Slowinski et al. [11] (Multicenter, 2015)	POS	General, surgical and medical ICUs	Former use of RCA; participation in other trials	LF	Circuit lifetime; metabolic complications; discharges status
Sponholz et al. [18] (GER, 2015)	ROS	Multidisciplinary ICU	NR	Liver transplantation	Acid-base status; electrolyte balance
Durao et al. [19] (BR, 2008)	ROS	ICU of tertiary care private hospital	NR	LF	Acid-base status; electrolyte balance
De Vico et al. [20] (IT, 2015)	ROS	Surgical ICU	NR	Cardiac surgery with LF	TEG value, episodes of hypocalcemia
Saner et al. [21] (GER, 2012)	ROS	Surgical ICU	Age < 18 years, incomplete records	Liver transplantation	Circuit lifetime; acid-base status; ionized calcium
Balogun et al. [22] (US, 2012)	ROS	NR	CRRT less than 24 h	End-stage liver disease	Circuit lifetime; metabolic complications; discharges status
Klingele et al. [23] (GER, 2017)	ROS	Interdisciplinary surgical ICU	ICG-PDR not performed	Cirrhosis; viral hepatitis; liver cancer	Metabolic disorders; predictors of citrate accumulation and metabolic alkalosis
Yu et al. [24] (CN, 2018)	ROS	ICU of an university hospital	CRRT less than 24 h	Acute LF	Circuit survival time; acid-base status; electrolyte balance; Lactate; blood pressure; TMP

*Abbreviations*: *AKI* acute kidney injury, *BR* Brazil, *CN* China, *CRRT* continuous renal replacement therapy, *GER* Germany, *IT* Italy, *ICU* intensive care unit, *ionCa* ionized calcium, *ICG-PDR* indocyanine green plasma disappearance rate, *LF* liver failure, *NR* not reported, *POS* prospective observational study, *ROS* retrospective observational study, *RCA* regional citrate anticoagulation, *TEG* thrombelastography, *TMP* trans-membrane pressure, *US* United States

**Table 2:** Characteristics of RCA-CRRT

Study	Type of CRRT	Device of CRRT	Citrate infusion	Calcium infusion	CRRT doses (ml/h)	Blood flow (ml/min)	Postfilter ionCa target (mmol/l)	Serum ionCa target (mmol/l)	Mean filter lifetime (h)
Schultheiss et al. [9]	CVVHD	HF440/Multifiltrate	4^ac^	1.7^be^	2000	100	0.25–0.35	1.12–1.20	NR
Lahmer et al. [10]	SLED	Genius system	Local formula	10^bd^	9000	150	0.35–0.45	1.00–1.10	NR
Slowinski et al. [11]	CVVHD	Multifiltrate	4^ac^	1.7^be^	NR	NR	0.25–0.35	1.12–1.20	71.1
Sponholz et al. [18]	CVVHD	Multifiltrate	4^ac^	1.7^be^	2000	120	0.25–0.35	NR	29 ± 42.2
Durao et al. [19]	CVVHDF	Prisma M100/AN69 filter	140^ad^	70^bd^	2000	100	0.25–0.30	1.12–1.20	72 ± 22.2
De Vico et al. [20]	CVVHDF	Prismaflex System	Local formula	6.38^bd^	NR	182	0.20–0.40	NR	49.76 ± 22.10
Saner et al. [21]	CVVHD	Fresenius ADM08/F60S dialyzer	Local formula	10% calcium gluconate	1000	75–100	< 0.30	> 0.95	22.7 ± 14.6
Balogun et al. [22]	NR	Prisma system/ST150 filter	180^d^	10^bd^	2000	180	NR	NR	62.4
Klingele et al. [23]	CVVHD	Multifiltrate-CiCa	4^ac^	1.7^be^	2000	100	0.25–0.35	NR	62.2 ± 11.2
Yu et al. [24]	NR	Gambro Prismaflex/AN69-M100 filter	NR	5%^b^	35 ml/h kg	3 ml/min·kg	0.25–0.45	1.0–1.2	44.2

*Abbreviations*: *CRRT* continuous renal replacement therapy, *CVVHD* continuous veno-venous hemodialysis, *CVVHDF* continuous veno-venous hemodiafiltration, *ionCa* ionized calcium, *NR* not reported, *RCA* regional citrate anticoagulation, *SLED* sustained low-efficiency dialysis

^a^4% trisodium citrate

^b^Calcium chloride

^c^mmol/l blood

^d^ml/h

^e^mmol/l

The patients’ baseline characteristics are showed in Table 3. Of the 1411 patients enrolled in the included studies, 170 had normal liver function and the remaining 1241 had different levels of liver dysfunction. The reported etiologies of liver dysfunction are the following: (i) decompensated liver cirrhosis, (ii) acute liver failure, (iii) perioperative liver transplantation, (iv) cardio-surgery, and (v) end-stage liver disease. The reported methods for evaluating the severity of liver failure include (i) the Model of End-stage Live Disease (MELD) score, (ii) total bilirubin (TB), (iii) Child-Pugh score, and (iv) prothrombin time index (PTI) ≤ 30%. Three (30%) studies [9, 10, 18] graded the severity of acute kidney injury (AKI) according to the Acute Kidney Injury Network classification (AKIN). The most common cause of AKI was sepsis (44.5%, 207/465 patients).

**Table 3:** Baseline data of the enrolled patients

Study	Sample size	Age (years)	F/M	MELD score	Total bilirubin (mg/dl)	Child-Pugh score	Creatinine (mg/dl)	pH	Serum ionized calcium (mmol/l)
Schultheiss et al. [9]	28	57 ± 11	8/20	36 ± 8.8	12 ± 14.7	12 ± 2.2	3.4 ± 2.0	7.29 ± 0.1	1.21 ± 0.08
Lahmer et al. [10]	24	59	4/20	35 ± 6.5	18.4 ± 14.5	≥ 10	3.8 ± 1.2	7.29 ± 0.05	1.14 ± 0.11
Slowinski et al. [11]	133	63 ± 15	38/95	NR	7.3	NR	2.8 ± 1.35	7.33	1.08 ± 0.12
Sponholz et al. [18]	89	54 ± 9.6	31/58	NR	7 ± 7.4	NR	2 ± 1.0	7.39 ± 0.07	1.11 ± 0.11
Durao et al. [19]	143	66 ± 16	59/84	NR	NR	NR	1.5 ± 0.88	7.29 ± 0.11	1.06 ± 0.06
De Vico et al. [20]	15	67.4 ± 11.9	5/10	NR	3.1 ± 3.38	NR	1.97 ± 0.88	7.38 ± 0.09	1.02 ± 0.06
Saner et al. [21]	68	47.1 ± 11.8	28/40	23.1 ± 9.1	8.7 ± 8.1	NR	2.35 ± 1.01	7.37 ± 0.08	1.08 ± 0.03
Balogun et al. [22]	697	56.4	281/416	17.7–44.81	11.4	NR	≥ 4	NR	NR
Klingele et al. [23]	69	59.1 ± 12.4	22/47	19.7 ± 9.6	9.5 ± 10.6	NR	2.7 ± 1.3	NR	NR
Yu et al. [24]	145	59 ± 17.7	92/53	NR	NR	NR	2.7 ± 3	NR	NR
Summarized	1411	57.9	568/843	25.2 ± 10.8	10.5	Grade C	2.44 ± 1.77	7.33 ± 0.1	1.08 ± 0.07

*Abbreviations*: *F* female, *M* male, *MELD* model of end-stage liver disease, *NR* not reported

### Quality evaluation

The results of the quality assessment of the 10 observational studies using the Newcastle-Ottawa Scale are described in Additional file 2: Table S2. Five studies [9, 11, 21–23] were scored 6–8 and were considered to be high-quality study. And the remaining 5 studies [10, 18–20, 24] were scored 4–5 and considered as moderate quality study.

### Efficacy

#### Filter lifespan

**Fig. 2:**
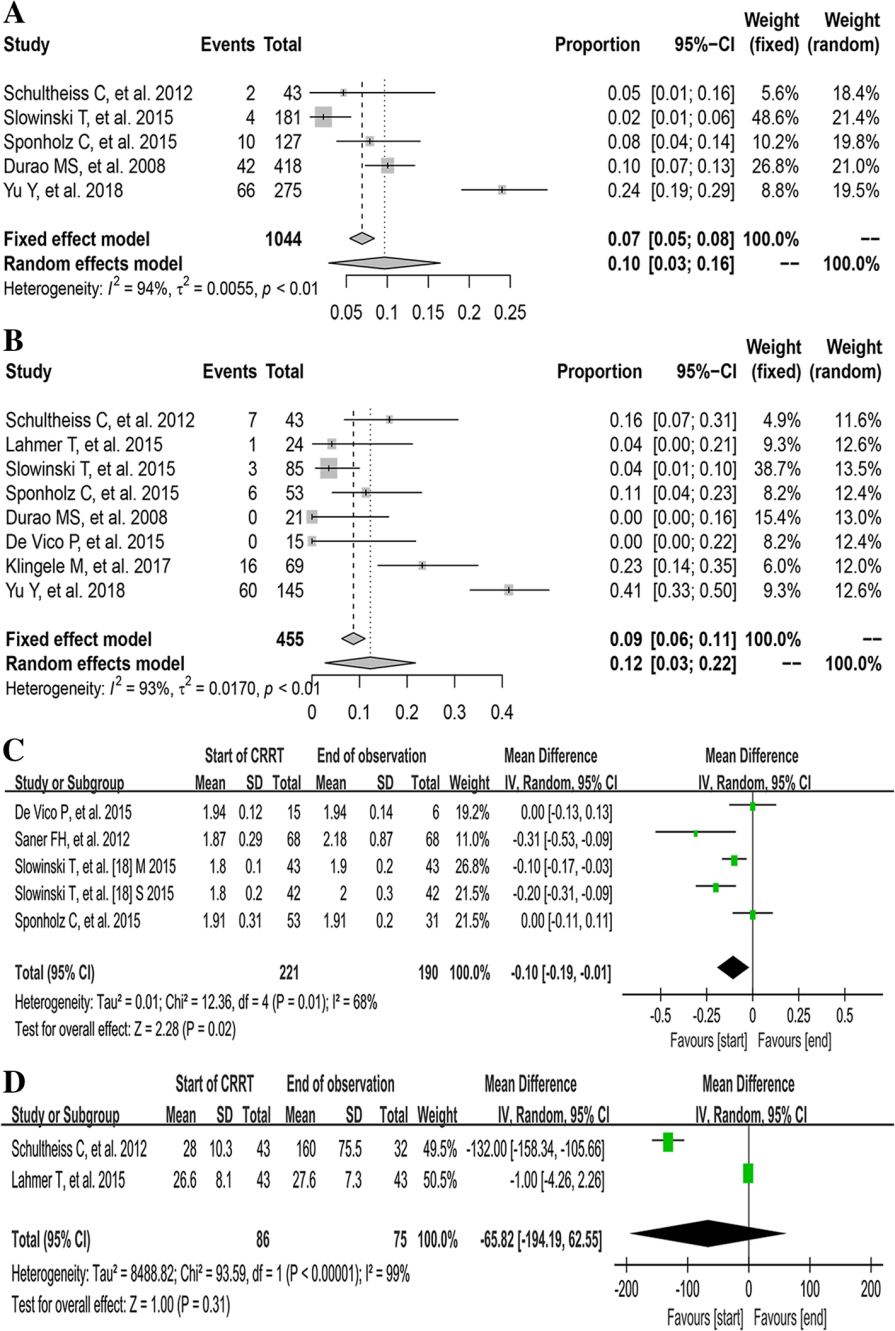
The pooled rates (95% CI) of filter clotting (**a**) and citrate accumulation (**b**), and the pooled MDs of totCa/ionCa ratio (**c**) and serum citrate level (**d**) between the start of CRRT and the end of observation. CI, confidence interval; ionCa, ionized calcium; MD, mean difference; M, mild liver failure group; S, Severe liver failure group; totCa, total calcium

Of the included studies, 8 [11, 18–24] reported the mean filter lifespan with a median of 55.9 h (IQR 32.8 to 68.9). And the minimum and maximum mean filter lifespan were 22.7 h [21] and 72 h [19], respectively (Table 2). The number of filters with clotting events were available in 5 studies [9, 11, 18, 19, 24], and the pooled rate of filter clotting was 10% (95% CI [3–16%]; *I*^2^ = 94%, *τ*^2^ = 0.0055, *P <* 0.01, Fig. 2a).

### Safety

#### Citrate accumulation

Eight studies [9–11, 18–20, 23, 24] reported the number of patients with citrate accumulation, which was identified by the increased ratio of totCa/ionCa. The pooled rate of citrate accumulation was 12% (95% CI [3–22%]; *I*^2^ = 93%, *τ*^2^ = 0.0170, *P <* 0.01, Fig. 2b). The mean serum level of totCa/ionCa ratio was available in four studies [11, 18, 20, 21]. The pooled result demonstrated that the totCa/ionCa ratio increased slightly during RCA-CRRT (MD − 0.10, 95% CI [− 0.19, − 0.01], *P =* 0.02; *I*^2^ = 68%, *P* = 0.01, Fig. 2c). The mean serum citrate concentration was available in two studies [9, 10]. And the pooled result demonstrated that no significant difference was observed between the start and the end of RCA-CRRT (MD − 65.82, 95% CI [− 194.19, 62.55], *P* = 0.31; *I*^2^ = 99%, *P* < 0.001, Fig. 2d).

#### Bleeding

**Fig. 3:**
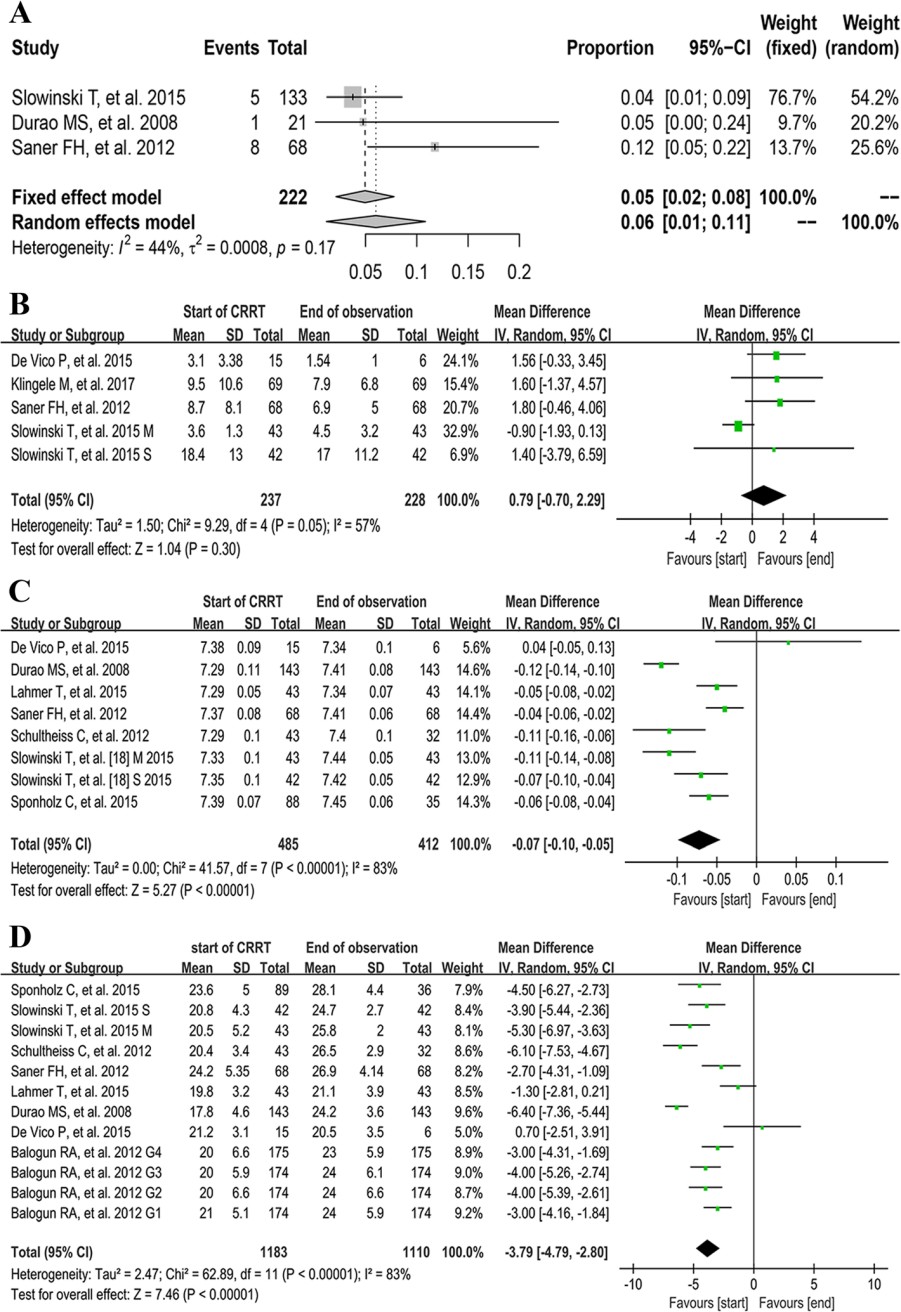
The pooled rates of bleeding (**a**) and the pooled MDs of total bilirubin (**b**), pH (**c**), and serum bicarbonate (**d**) between the start of CRRT and the end of observation. CI, confidence interval; G1 group 1, G2 group 2, G3 group 3, G4 group 4; MD, mean difference; M, mild liver failure group; S, Severe liver failure group

Three studies [11, 19, 21] reported the number of patients with bleeding, and the pooled rate of bleeding was 5% (95% CI [2–8%]; *I*^2^ = 44%, *τ*^2^ = 0.0008, *P =* 0.17, Fig. 3a).

#### Total bilirubin

The data of total bilirubin were available in four studies [11, 20, 21, 23]. The pooled result demonstrated that there were no significant difference in total bilirubin between the start and the end of RCA-CRRT (MD 0.79, 95% CI [− 0.70, 2.29], *P* = 0.30; *I*^2^ = 57%, *P =* 0.05, Fig. 3b).

#### Acid-base status at the start of CRRT versus the end of observation

The pooled results demonstrated that the pH (MD − 0.07, 95% CI [− 0.10, − 0.05], *P <* 0.001; *I*^2^ = 83%, *P <* 0.001, Fig. 3c), serum bicarbonate (MD − 3.79, 95% CI [− 4.79, − 2.80], *P <* 0.001; *I*^2^ = 83%, *P <* 0.001, Fig. 3d), and BE (MD − 4.57, 95% CI [− 6.46, − 2.68], *P <* 0.001; *I*^2^ = 90%, *P <* 0.001, Additional file 3: Figure S1A) increased significantly during RCA-CRRT. And the rate of metabolic alkalosis increased by 38% (RD − 0.38, 95% CI [− 0.48, − 0.28], *P* < 0.001; *I*^2^ = 44%, *P =* 0.18) at the end of RCA-CRRT (Additional file 3: Figure S1B). The pooled rate of metabolic alkalosis was 13% (95% CI [0–35%]; *I*^2^ = 94%, τ^2^ = 0.0233, *P <* 0.01, Additional file 3: Figure S1C). No significant change was observed in metabolic acidosis rate (RD 0.25, 95% CI [− 0.25, 0.75], *P* = 0.33; *I*^2^ = 97%, *P <* 0.001, Additional file 3: Figure S1D) and serum lactate (MD 0.49, 95% CI [− 0.27, 1.26], *P* = 0.21; *I*^2^ = 66%, *P =* 0.02, Additional file 4: Figure S2A) at the end of observation.

#### Serum calcium at the start of CRRT versus the end of observation

As shown in Additional file 4: Figure S2B-C, both the ionCa (MD − 0.07, 95% CI [− 0.11, − 0.03], *P* = 0.002; *I*^2^ = 93%*, P <* 0.001) and totCa (MD − 0.30, 95% CI [− 0.45, − 0.15], *P* < 0.001; *I*^2^ = 96%*, P* < 0.001) in the systemic circulation increased significantly at the end of RCA-CRRT. The rate of hypocalcemia was similar between the start and end of CRRT (RD 0.06, 95% CI [− 0.19, 0.31], *P* = 0.62; *I*^2^ = 83%*, P* = 0.003, Additional file 4: Figure S2D).

#### Liver failure patients versus non-liver failure patients

Two included studies [19, 24] compared the pH, serum lactate, and totCa/ionCa ratio between liver failure patients and non-liver failure patients. The pooled results demonstrated that there were no significant differences in pH (MD -0.04, 95% CI [− 0.13, 0.05], *P =* 0.34; *I*^2^ = 93%, *P <* 0.001), serum lactate (MD 0.69, 95% CI [− 0.26, 1.64], *P* = 0.16; *I*^2^ = 58%, *P =* 0.13), and totCa/ionCa ratio (MD 0.03, 95% CI [− 0.12, 0.18], *P* = 0.69; *I*^2^ = 84%, *P =* 0.01) between the two groups (Additional file 5: Figure S3A-C).

#### Risk factors of citrate accumulation and circuit lifespan

There were three studies reported the risk factors of citrate accumulation. One study [9] identified that baseline serum lactate > 3.4 mmol/l and pro-thrombin time < 26% were independent risk factors of citrate accumulation. And the elevated citrate level before the start of dialysis was reported as one of the risk factors by another study [10]. However, the remaining study [24] identified no risk factor of the increased totCa/ionCa ratio. None of the 10 included studies conducted multivariate or univariate analysis to identify the risk factors of circuit lifespan.

## Discussion

It is controversial on the use of RCA for CRRT in patients with liver failure. Our systematic review has several findings on this field. First, RCA during CRRT did not significantly increase the risk of citrate accumulation in patients with liver dysfunction compared with the patients without liver dysfunction. Second, the acid-base status time trend was from the acidotic range towards alkaline during RCA-CRRT. Third, serum calcium increased slightly and the rate of calcium disarrangement was similar between the start and the end of CRRT. Fourth, the liver failure patients had comparable pH, serum lactate, and totCa/ionCa ratio during RCA-CRRT, compared with non-liver failure patients. At last, in liver failure patients, the filter lifespan of RCA-CRRT was prolonged significantly. These findings could provide clinicians helpful information on the use of RCA in liver failure patients requiring CRRT.

Patients with liver dysfunction are prone to coagulopathy that may contribute to extended CRRT filter lifespan [25]. However, some researches proved that patients with liver dysfunction have a parallel decrease in both procoagulant and anticoagulant factors which leads to coagulation system disorder, which means they could have either a hypo- or hypercoagulable situation [26, 27]. Most likely, liver failure patients underwent CRRT are at high risk of both bleeding and filter clotting. The reported mean filter lifespan of liver failure patients underwent CRRT without anticoagulation ranged from 7.4 to 12 h [28, 29]. In our systematic review, the pooled median filter lifespan of RCA-CRRT were significantly longer than the reported filter lifespan in CRRT without anticoagulation. And the filter lifespan of RCA-CRRT in liver failure patients was comparable with the previous reported filter lifespan of RCA-CRRT in non-liver failure patients, which was ranged from 24.2 to 82 h (median 48 h, IQR 26.85–76 h) [30–34].

In a meta-analysis, Liu et al. [2] demonstrated that the pooled incidence of bleeding in RCA-CRRT groups was 4.2% in patients without liver failure. In a randomized controlled study, Stucker et al. [35] reported that the incidence of clotting events in RCA-CRRT filters was 6% in no-liver failure patients. Our results suggested that patients with liver failure and those without might have comparable risk of bleeding and filter clotting.

During RCA-CRRT, about 30–70% of the administrated citrate could be removed by dialyzer, and the remaining citrate enters the systemic circulation [36, 37]. In the setting of severe liver dysfunction, citrate clearance is reduced by about 50%, which means liver failure patients are more susceptible to citrate accumulation [3, 5]. Khadzhynov et al. [38] reported that citrate accumulation occurred in 32 (2.99%) out of 1070 patients underwent RCA-CRRT. And 11 of the 32 cases (34%) had pre-existing liver dysfunction. In our present systematic review, the pooled citrate accumulation rate in liver failure patients is 12% (95% CI 3–22%), which is higher than the previously reported incidence of citrate accumulation in total patients.

However, we found out that most of the 8 studies [9–11, 18–20, 23, 24] in our systematic review identified citrate accumulation only by the increased totCa/ionCa ratio. Based on this criterion, Meier-Kriesche et al. [4] reported that citrate accumulation occurred in 33% patients with liver failure. Khadzhynov et al. [38] considered that the ionized hypocalcemia, which could be induced by other causes, was not specific enough for the diagnosis of citrate accumulation and that the increased totCa/ionCa ratio could not predict citrate accumulation in all cases. Therefore, Khadzhynov et al. [38] recommended more rigorous diagnosis criteria for citrate accumulation: (i) decreased systemic ionized calcium, (ii) increased demand for calcium substitution, (iii) elevated totCa/ionCa ratio, and (iv) metabolic acidosis. Most likely, the citrate accumulation incidences of the included studies were over-estimated by only using the totCa/ionCa ratio.

Theoretically, liver failure patients did not loss all of the liver citrate metabolization function and preserved the ability of metabolizing citrate in the skeletal muscle and kidney cortex [39]. Most likely, with carefully monitoring the serum totCa/ionCa level and timely adjusting CRRT model and citrate dose, citrate accumulation could be additionally reduced in liver failure patients.

The pooled results demonstrated that the distribution of pH, serum bicarbonate concentration, and BE shifted from the acidotic range towards alkaline range during RCA-CRRT. This phenomenon has been well explained in the study by Schneider et al. [40]. In this study, the authors considered the development of alkalosis in RCA-CRRT as citrate overload. Commonly, the citrate overload could be corrected by the reduction of citrate delivery without the change of anticoagulant for CRRT. Additionally, most of the included studies used the replacement solution and dialysates with fixed bicarbonate concentration. RCA-CRRT could lead to plasma alkalization due to the metabolism of citrate [1, 40]. Based on the additional bicarbonate load during RCA-CRRT, the bicarbonate concentration should be relevantly reduced to avoid the occurrence of alkalosis. Mehta et al. [41] and Morgera et al. [42] reported that the incidences of metabolic alkalosis in non-liver failure patients who received RCA-CRRT were 23% and 50%, respectively. The significant heterogeneity of metabolic alkalosis rate was also observed in our systematic review in liver failure patients, which most likely due to the variation of CRRT protocols.

Of the 10 included studies, only one study [11] reported that 13 (15%) out of 85 liver failure patients developed new episodes of metabolic acidosis during RCA-CRRT. However, half of these patients had preexisting acidosis at the initiation of CRRT. In the remaining 9 studies, the incidence of metabolic acidosis was not clearly reported. Therefore, in further studies on RCA-CRRT in liver failure patients, more attention should be paid to the occurrence of metabolic acidosis.

Citrate anticoagulation might cause severe hypocalcemia, especially when citrate metabolism was impaired due to liver dysfunction [4, 43]. Severe systemic ionized hypocalcemia is a life-threatening complication, which may lead to weakness, myocardial dysfunction, and death [44, 45]. However, the pooled results demonstrated that the ionCa and totCa levels were significantly increased at the end of CRRT. Additionally, the pooled incidences of hypocalcemia at the start and end of CRRT were not significantly different. These results suggest that RCA-CRRT performed under the guidance of an appropriate protocol most likely does not increase the risk of hypocalcemia in liver failure patients.

For the potential increased risks of citrate-related complications in liver failure patients, all of the included studies had employed some individualized prophylactic methods (Additional file 6: Table S3). However, further studies are needed to evaluate the efficacy of these methods on the reduction of citrate-related complications.

There are some limitations in our systematic review. First, we have not evaluated the potential publication bias by funnel plots. It is reported that funnel plots generally are less useful in the context of observational meta-analyses [46], and tests for funnel plot asymmetry are not recommended for meta-analysis with less than 10 studies [47]. Second, the etiologies and severity of liver failure, CRRT models, and RCA protocols are varied across the included studies. Factors affected the acid-base status and electrolyte balance may be complicated by the variations of these important characteristics [48]. Third, significant heterogenicity was observed among the included studies, with *I*^2^ value ranged from 44 to 99%. We have conducted sensitivity and subgroup analyses to try to find the cause of heterogenicity. However, all of these work failed to identify any explanation for the significant heterogenicity. At last, all of the included studies were observational studies and majority of them did not have control group. In order to present a stronger conclusion, we pooled the results of the comparisons between the start and end of observation and the comparisons between the patients with different liver function. Therefore, further well-designed studies are warranted to evaluate the safety and efficacy of citrate for CRRT in liver failure patients. Accordingly, we are performing a retrospective study and a randomized controlled trial to evaluate the efficacy of regional citrate anticoagulation versus no-anticoagulation for CRRT in patients with liver failure and increased bleeding risk.

## Conclusion

RCA-CRRT might be safe and effective in liver failure patients with a prolonged filter lifespan. The increased risk of citrate accumulation is the major limitation of RCA-CRRT, which most likely could be well addressed by careful monitoring and timely strategy-adjusting. Intensive monitor of the acid base status and calcium parameters may be more necessary during RCA-CRRT in patients with liver failure. Further studies with large sample size, control group, prospective design, uniformed standards, and randomized assignment to the intervention groups are needed to provide higher quality evidences on the anticoagulation for CRRT in liver failure patients.

## Additional files

Additional file 1: **Table S1.** The Modified version of the Newcastle-Ottawa Scale for assessing the quality of nonrandomized studies in meta-analyses. (DOCX 26 kb)

Additional file 2: **Table S2.** Methodological quality of the included studies. (DOCX 20 kb)

Additional file 3: **Figure S1.** The pooled MD of BE (A), the pooled rates of metabolic alkalosis (B), and the pooled RD of metabolic alkalosis (C) and acidosis rate (D) between the start of CRRT and the end of observation. BE, base excess; CI, confidence interval; MD, mean difference; M, mild liver failure group; RD, risk difference; S, Severe liver failure group. (TIF 6718 kb)

Additional file 4: **Figure S2.** The pooled MD of serum lactate (A), serum ionized calcium (B) and total calcium (C), and the pooled RD of ionized hypocalcemia (D) between the start of CRRT and the end of observation. All the results were demonstrated in forest plot. CI, confidence interval; G1 group 1, G2 group 2, G3 group 3, G4 group 4; MD, mean difference; M, mild liver failure group; RD, risk difference; S, Severe liver failure group. (TIF 5817 kb)

Additional file 5: **Figure S3.** The pooled MD of pH (A), serum lactate (B) and totCa/ionCa ratio (C) between the liver failure patients and non-liver failure patient. CI, confidence interval; ionCa, ionized calcium; MD, mean difference; totCa, total calcium; LF, liver failure. (TIF 2930 kb)

Additional file 6: **Table S3.** Management of metabolic complications and citrate accumulation. (DOCX 20 kb)

## Abbreviations

AKI: Acute kidney injury
AKIN: Acute Kidney Injury Network classification
BE: Base excess
BR: Brazil
CI: Confidential interval
CN: China
CRRT: Continuous renal replacement therapy
CVVHD: Continuous veno-venous hemodialysis
CVVHDF: Continuous veno-venous hemodiafiltration
F: Female
GER: Germany
ICG-PDR: Indocyanine green plasma disappearance rate
ICU: Intensive care unit
ionCa: Ionized calcium
IQR: Inter-quartile range
IT: Italy
KDIGO: Kidney Disease Improving Global Outcomes
LF: Liver failure
M: Male
MD: Mean difference
MELD: Model of end-stage liver disease
NR: Not reported
POS: Prospective observational study
PTI: Prothrombin time index
RCA: Regional citrate anticoagulation
RD: Risk difference
ROS: Retrospective observational study
SD: Standard deviation
SLED: Sustained low-efficiency dialysis
TB: Total bilirubin
TEG: Thrombelastography
TMP: Trans-membrane pressure
totCa: Total calcium
US: United States
