# Supplementary material for: Safety and efficacy of regional citrate anticoagulation for continuous renal replacement therapy in liver failure patients: a systematic review and meta-analysis
Source: Crit Care. 2019 Jan 24;23:22. doi: 10.1186/s13054-019-2317-9 (PMC6345001; doi:10.1186/s13054-019-2317-9)
Supplement: Supplementary file 1 — Table S1. The Modified version of the Newcastle-Ottawa Scale for assessing the quality of nonrandomized studies in meta-analyses. (DOCX 26 kb) [file 13054_2019_2317_MOESM1_ESM.docx]

| **Table S1. The modified version of the Newcastle-Ottawa Scale for assessing the quality of non-randomized studies in meta-analyses** | | | | |
| --- | --- | --- | --- | --- |
| Quality-assessed Items | Sub-items | | Criteria and scores | |
| **Subjects selection** | **Selection 1** | Representativeness of the liver failure patients included in the studies | a | Truly representative of the general characteristics of liver failure patients (1 score) |
|  |  |  | b | Somewhat representative of the general characteristics of the liver failure patients (1 score) |
|  |  |  | c | Selected from some population eg general ICU patients (0 score) |
|  |  |  | d | No description of the derivation of the cohort (0 score) |
|  | **Selection 2** | Selection of the non liver failure patients | a | Drawn from the same ICU of liver failure patients (1 score) |
|  |  |  | b | Drawn from a different source (1 score) |
|  |  |  | c | No non-liver failure patients were enrolled (0 score) |
|  | **Selection 3** | Ascertainment of liver failure | a | Secure record (eg electronic medical records, surgical records) (1 score) |
|  |  |  | b | Structured interview (1 score) |
|  |  |  | c | Written self report (0 score) |
|  |  |  | d | No description (0 score) |
|  | **Selection 4** | Demonstration that endpoint events were not presented at start of study | a | Yes (1 score) |
|  |  |  | b | No (0 score) |
| **Comparability** | Comparability of cohorts on the basis of the design or analysis | | a | Study controls for liver failure severity (1 score) |
|  |  |  | b | Study controls for any additional factor (1 score) |
|  |  |  | c | None (0 score) |
| **Outcome** | **Outcome 1** | Assessment of outcome | a | Independent blind assessment (1 score) |
|  |  |  | b | Record linkage (1 score) |
|  |  |  | c | Self report (0 score) |
|  |  |  | d | No description (0 score) |
|  | **Outcome 2** | Was observation long enough for endpoint events to occur | a | Yes (select an adequate observational period for events of interest) (1 score) |
|  |  |  | b | No (0 score) |
|  | **Outcome 3** | Completeness of the observation period | a | Follow up reach the predefined observation endpoint - all subjects accounted for (1 score) |
|  |  |  | b | Subjects lost to follow up unlikely to introduce bias - small number lost < 10% (1 score) |
|  |  |  | c | Subjects lost to follow up > 25% (0 score) |
|  |  |  | d | No statement (0 score) |
| Note: A study can score a maximum of 1 for each numbered item within the Selection and Outcome categories. A maximum of 2 can be given for Comparability. ICU, intensive care unit. | | | | |
