# Supplementary material for: Safety and efficacy of regional citrate anticoagulation for continuous renal replacement therapy in liver failure patients: a systematic review and meta-analysis
Source: Crit Care. 2019 Jan 24;23:22. doi: 10.1186/s13054-019-2317-9 (PMC6345001; doi:10.1186/s13054-019-2317-9)
Supplement: Supplementary file 2 — Table S2. Methodological quality of the included studies. (DOCX 20 kb) [file 13054_2019_2317_MOESM2_ESM.docx]

| **Table S2. Methodological quality of the included studies** | | | | | | | | | |
| --- | --- | --- | --- | --- | --- | --- | --- | --- | --- |
| **Author, Year Study Design** | **Selection 1** | **Selection 2** | **Selection 3** | **Selection 4** | **Comparability** | **Outcome 1** | **Outcome 2** | **Outcome 3** | **Total scores** |
| Schultheiss C, et al., 2012  POS | A | C | A | B | B | B | A | A | 6 |
| Lahmer T, et al., 2015  POS | A | C | A | B | C | B | A | A | 5 |
| Slowinski T, et al., 2015  POS | A | A | A | B | AB | B | A | A | 8 |
| Sponholz C, et al., 2015  ROS | A | C | A | B | C | B | A | C | 4 |
| Durao MS, et al., 2008  ROS | B | A | A | B | C | B | B | A | 5 |
| De Vico P, et al., 2015  ROS | B | C | A | B | C | B | A | C | 4 |
| Saner FH, et al., 2012  ROS | A | C | A | B | B | B | A | A | 6 |
| Balogun RA, et al., 2012  ROS | A | C | A | B | A | B | A | A | 6 |
| Klingele M, et al., 2017  ROS | A | C | A | B | B | B | A | A | 6 |
| Yu Y, et al., 2018  ROS | B | A | A | B | A | B | A | C | 5 |
| Abbreviations: POS, prospective observational study; ROS, retrospective observational study. | | | | | | | | | |
