# Supplementary figures and images for: Safety and efficacy of regional citrate anticoagulation for continuous renal replacement therapy in liver failure patients: a systematic review and meta-analysis
Source: Crit Care. 2019 Jan 24;23:22. doi: 10.1186/s13054-019-2317-9 (PMC6345001; doi:10.1186/s13054-019-2317-9)

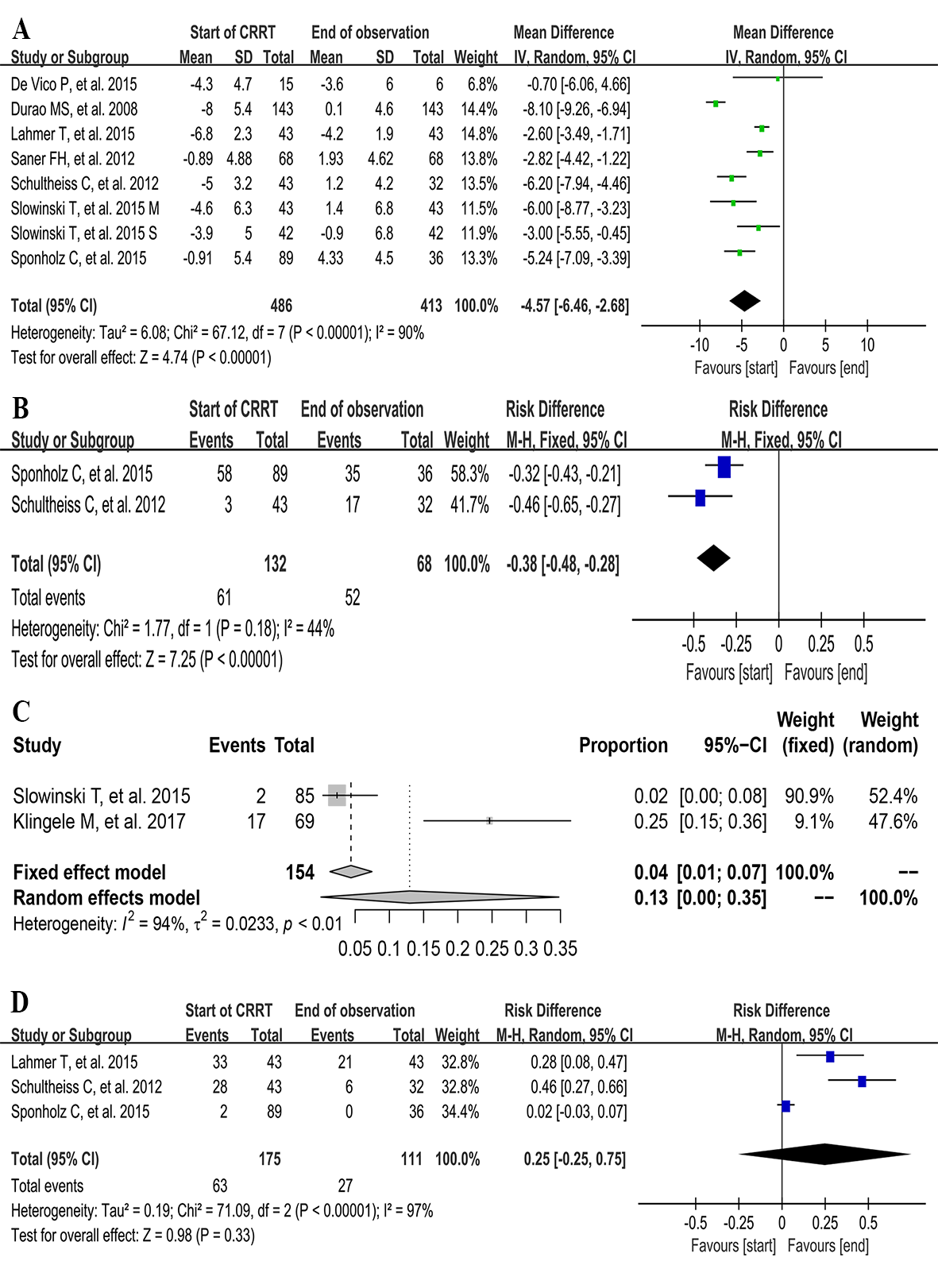

Supplement: Supplementary file 3 — Figure S1. The pooled MD of BE (A), the pooled rates of metabolic alkalosis (B), and the pooled RD of metabolic alkalosis (C) and acidosis rate (D) between the start of CRRT and the end of observation. BE, base excess; CI, confidence interval; MD, mean difference; M, mild liver failure group; RD, risk difference; S, Severe liver failure group. (TIF 6718 kb) [file 13054_2019_2317_MOESM3_ESM.tif]

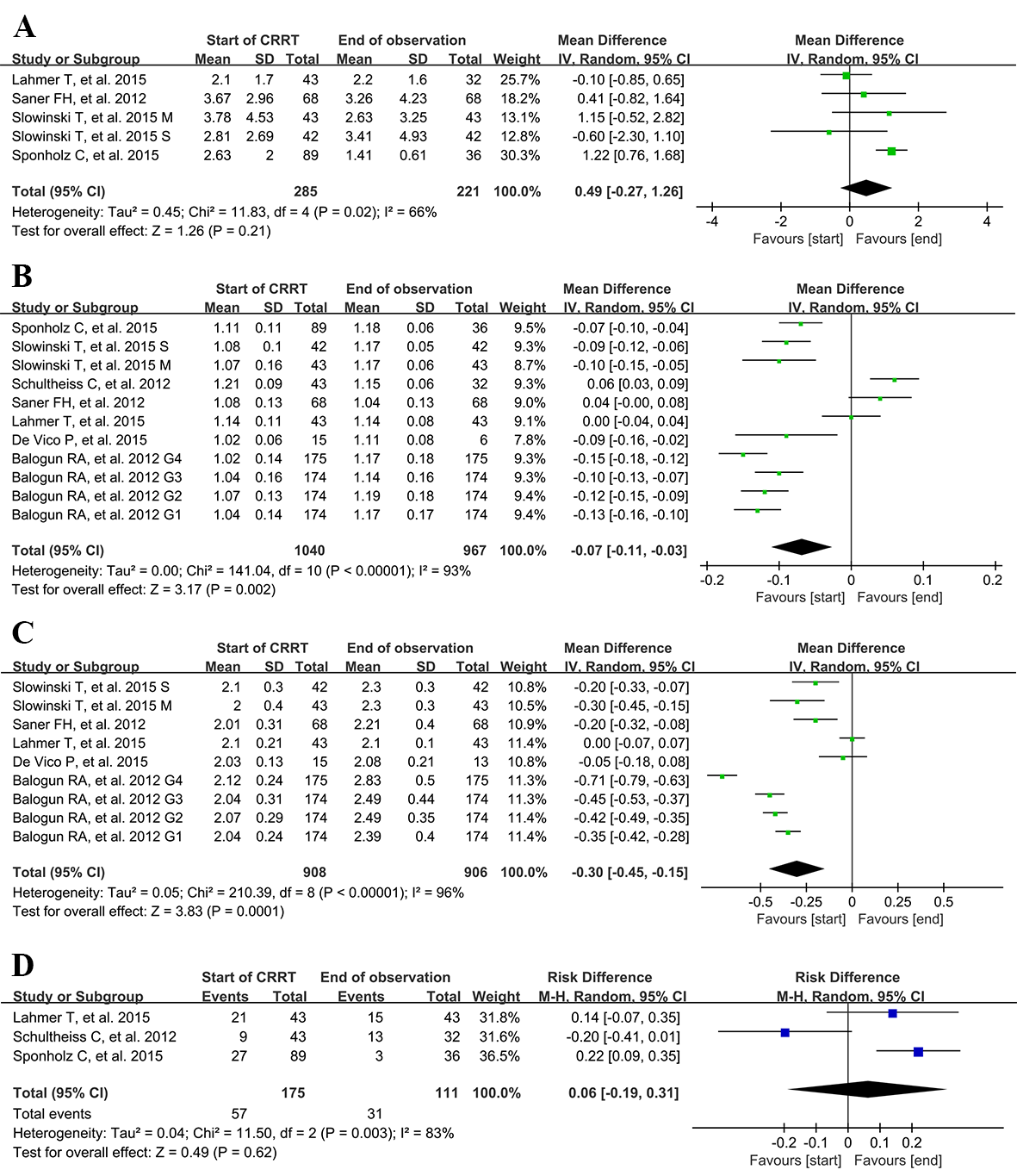

Supplement: Supplementary file 4 — Figure S2. The pooled MD of serum lactate (A), serum ionized calcium (B) and total calcium (C), and the pooled RD of ionized hypocalcemia (D) between the start of CRRT and the end of observation. All the results were demonstrated in forest plot. CI, confidence interval; G1 group 1, G2 group 2, G3 group 3, G4 group 4; MD, mean difference; M, mild liver failure group; RD, risk difference; S, Severe liver failure group. (TIF 5817 kb) [file 13054_2019_2317_MOESM4_ESM.tif]

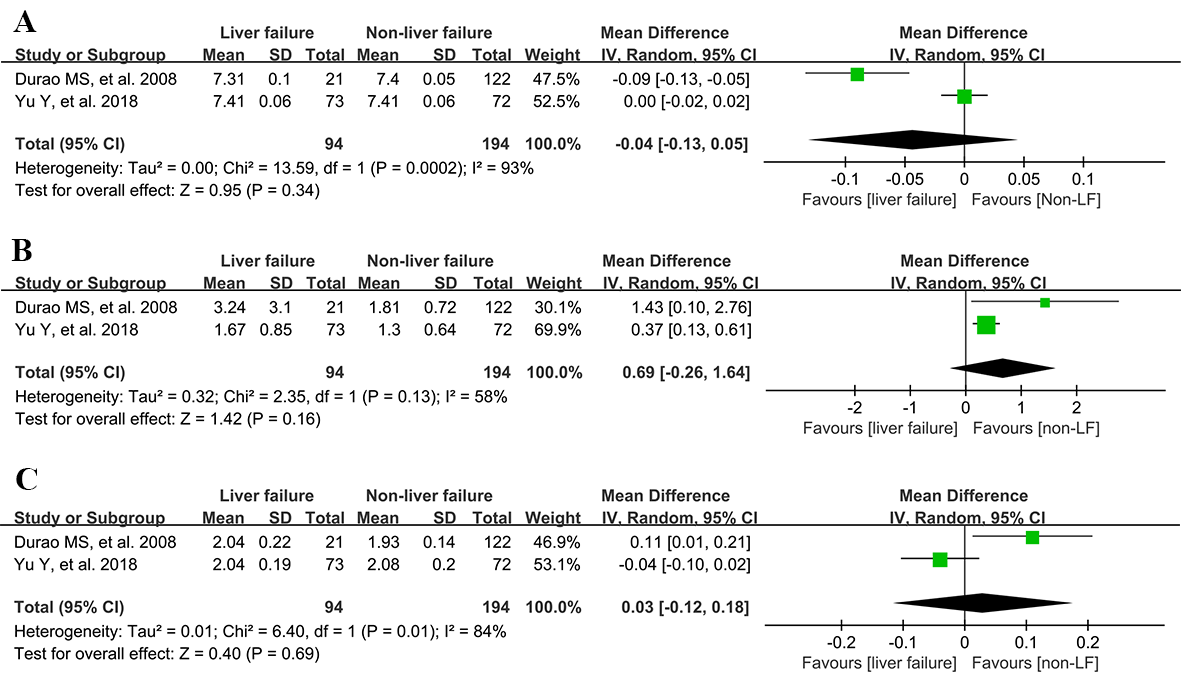

Supplement: Supplementary file 5 — Figure S3. The pooled MD of pH (A), serum lactate (B) and totCa/ionCa ratio (C) between the liver failure patients and non-liver failure patient. CI, confidence interval; ionCa, ionized calcium; MD, mean difference; totCa, total calcium; LF, liver failure. (TIF 2930 kb) [file 13054_2019_2317_MOESM5_ESM.tif]
