# Supplementary material for: Safety and efficacy of regional citrate anticoagulation for continuous renal replacement therapy in liver failure patients: a systematic review and meta-analysis
Source: Crit Care. 2019 Jan 24;23:22. doi: 10.1186/s13054-019-2317-9 (PMC6345001; doi:10.1186/s13054-019-2317-9)
Supplement: Supplementary file 6 — Table S3. Management of metabolic complications and citrate accumulation. (DOCX 20 kb) [file 13054_2019_2317_MOESM6_ESM.docx]

| Table S3. Management of metabolic complications and citrate accumulation | |
| --- | --- |
| Study | Prophylactic methods |
| Schultheiss C, *et al.* [9] | 1. Careful monitoring of electrolytes and acid-base status; 2. Regularly monitoring ionCa in the circuit and patient’s circulation during the treatment. |
| Lahmer T, *et al.* [10] | 1. regularly monitor ionCa in the patients’ circulation during treatment; 2. Continuously monitor the acid-base status and calcium parameters. |
| Slowinski T, *et al.* [11] | 1. In case of severe alkalosis, adjust (increase) the ratio of dialysate to blood flow; 2. In case of hypocalcemia, adapt calcium supplementation. |
| Sponholz C, *et al.* [18] | 1. Modify dialysis protocols; 2. Adjust citrate or dialysis solutions. |
| Durao MS, *et al.* [19] | 1. Assessed plasma citrate levels ; 2. limit citrate infusion; 3. Allow higher levels of postfilter ionized calcium concentrations; 4. Increase effluent flow (dialysis dose). |
| De Vico P, *et al.* [20] | Use low concentration citrate-based solution and calcium-free dialysate. |
| Saner FH, *et al.* [21] | 1. Change the solute concentration and flow rate of dialysate; 2. Use dialysate containing calcium; 3. Strict monitoring of acid-base status. |
| Balogun RA, *et al.* [22] | 1. Reduce citrate infusion rates; 2. Carefully monitor systemic ionized calcium. |
| Klingele M, *et al.* [23] | 1. In case of citrate accumulation, dialysate flow was increased and blood flow was reduced; 2. Use dialysate solution with reduced bicarbonate content; 3. Close monitoring of metabolic disorders. |
| Yu Y, *et al.* [24] | Proper monitoring and adjustment of citrate and calcium infusion. |
| Abbreviation: ionCa, ionized calcium. | |
